# Uncomplicated Diverticulosis Is Not Associated With Abdominal Pain or Abnormal Bowel Habit—A Population‐Based Swedish Cohort Study

**DOI:** 10.1111/nmo.70000

**Published:** 2025-02-17

**Authors:** Bjarki T. Alexandersson, Michael P. Jones, Anna Forsberg, Charlotte R. H. Hedin, Ellionore Järbrink‐Sehgal, Susanna Walter, Nicholas J. Talley, Lars Agreus, Anna Andreasson, Peter T. Schmidt

**Affiliations:** ^1^ Department of Medicine Solna Karolinska Institutet Stockholm Sweden; ^2^ School of Psychological Sciences Macquarie University North Ryde Australia; ^3^ Department of Gastroenterology, Dermatovenereology and Rheumatology Centre for Digestive Health, Karolinska University Hospital Stockholm Sweden; ^4^ Gastroenterology, Hepatology and Nutrition Department UT MD Anderson Cancer Center Houston Texas USA; ^5^ Department of Health, Medicine and Caring Sciences (HMV) Linköping University Linköping Sweden; ^6^ School of Medicine and Public Health University of Newcastle Newcastle New South Wales Australia; ^7^ Department of Neurobiology, Care Sciences and Society Karolinska Institutet Huddinge Sweden; ^8^ Department of Psychology Stockholm University Stockholm Sweden; ^9^ Department of Medical Sciences Uppsala University Uppsala Sweden

**Keywords:** abdominal pain, colonoscopy, diverticular diseases, diverticulitis, irritable bowel syndrome, surveys and questionnaires

## Abstract

**Background:**

Whether uncomplicated diverticulosis gives rise to symptoms is controversial. Diary‐based studies of abdominal pain and stool habits in general populations are scarce, and we therefore investigated symptom patterns in diverticulosis from prospectively collected symptom diaries in a random sample of the general population who completed a research colonoscopy.

**Methods:**

In the Swedish population‐based colonoscopy (PopCol) study, 745 individuals from the general population underwent a colonoscopy of which 130 had diverticulosis, and none had diverticulitis. Seven‐day symptom diaries were completed by 258 participants (age 54, women 64%) of which 50 had diverticulosis. The frequency and location of abdominal pain, bowel habit and other gastrointestinal symptoms were compared between individuals with and without diverticulosis using logistic regression.

**Key Results:**

Diverticulosis was not associated with abdominal pain (OR 1.24, CI 0.61–2.55) or left lower quadrant (LLQ) abdominal pain (OR 1.59, CI 0.73–3.49). Pain duration and severity were not associated with diverticulosis. When individuals with diverticulosis had pain, it was more often in the LLQ (OR 2.45, CI 1.02–5.86) compared with those without diverticulosis. Diverticulosis was not linked to altered bowel habits. Irritable bowel syndrome prevalence was 16% in the diverticulosis group and 19% in the non‐diverticulosis group.

**Conclusions and Inferences:**

Participants with diverticulosis did not report more abdominal pain or more LLQ abdominal pain than participants without diverticulosis. Bowel habit was not abnormal in diverticulosis. Our results do not support that uncomplicated diverticulosis cause symptoms in individuals without a history of acute diverticulitis.

## Graphical abstract

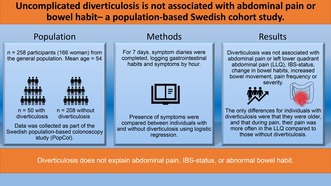

**Summary**

- Whether individuals with diverticulosis without any complications have increased abdominal pain is unclear and real time diary‐based studies are lacking.
- In our results individuals with diverticulosis do not have more abdominal pain than those without diverticulosis nor do they have different bowel habits.
- Thus diverticulosis does not explain abdominal pain or changes in bowel habits.

## 1 Introduction

Diverticulosis is one of the most frequent findings on colonoscopy with a higher prevalence in older individuals [1]. The mucosa protrudes between the muscular layers at vascular entry sites forming a pouch like structure termed a diverticulum [2]. Complications of diverticulosis include diverticular hemorrhage, acute diverticulitis, and segmental colitis associated with diverticulosis. Whether uncomplicated diverticulosis gives rise to symptoms is controversial. Some studies suggest that individuals with diverticulosis have more severe and more prolonged pain than those without it [3]. However other studies indicate that there is no difference in pain frequency between the two groups [4]. One theory is that pain associated with diverticulosis is a subset of irritable bowel syndrome (IBS) [5] but there are indications that diverticulosis pain differs from pain in IBS and may therefore be a distinct clinical entity [3, 6, 7, 8, 9]. We have previously studied symptoms and bowel habits in the Swedish population‐based colonoscopy study (PopCol) using questionnaires and found that individuals with diverticulosis did not report more abdominal pain on questionnaires than those without diverticulosis but had more loose stools [10]. However, the questionnaires did not consider the localization of pain and were based on recall which has been shown to be unreliable [11].

The present study sought to investigate the frequency, intensity, and location of abdominal pain and bowel habits using prospective abdominal symptom diaries from the PopCol study comparing participants with and without diverticulosis. The hypothesis tested was that participants with diverticulosis would report more abdominal pain and that the pain more often would be in the LLQ compared with those without diverticulosis.

## 2 Methods and Materials

This population‐based cohort study is a part of the Swedish population‐based colonoscopy study (PopCol), performed between 2002 and 2006, aiming to investigate gastrointestinal (GI) symptoms and pathology in the general population. A detailed description of participant recruitment for the study as well as its methodology has been previously described [12]. The study was approved by the local ethics committee at Karolinska Institutet (D.nr. 394/01).

### 2.1 Study Population

A random sample of 3556 individuals (aged 18–70 years) from Maria and Katarina parishes, two adjacent urban districts in Stockholm, Sweden were invited to participate in the study. Individuals born outside of Sweden were excluded. The study region is socio‐demographically similar to Sweden overall, with the mean income comparable to the mean income in Sweden although education level was higher in the two urban districts. Of 2293 respondents, 1673 were reached by telephone, of which 1244 were scheduled for a gastroenterologist consultation. The gastroenterologist was blinded to the questionnaire reports. A total of 745 participants agreed to undergo a research colonoscopy. Seven‐day symptom diaries [13] were completed by 272 participants. Four had missing data or an organic gastrointestinal disease and 10 did not undergo colonoscopy leaving 258 as the final study group. No one had a previous history of acute diverticulitis. Compared to the original study cohort of 3556 individuals the study group was slightly older (mean age 53.8 compared with 48.7), and females were slightly overrepresented (64.2% compared with 57.3%). However, there were no differences between groups according to the abdominal symptoms and bowel habits on the abdominal symptom questionnaire.

### 2.2 Diverticulosis

The endoscopists were instructed to record all pathological findings with localization. Of the 258 in the study group 50 had one or more diverticula (19%), of which all had involvement of the sigmoid colon. Of those that had one or more diverticula 20% also had involvement in the descending colon, 12% in transverse colon, 4% in ascending colon, and 4% in the cecum.

### 2.3 Symptom Diaries

The symptom diaries were completed prior to the colonoscopy, before bowel cleaning was commenced and filled in for every hour for 7 consecutive days. Each hour with pain was reported and the severity, location, and duration of abdominal pain was registered for each episode. Pain severity was registered on a three‐point scale from mild pain (1) to moderate (2) and severe pain (3). Location of the abdominal pain was registered by the participant on a diagram of the abdomen. The participants' location of pain was later mapped onto four quadrants. We defined LLQ pain as all pain occasions that involved the LLQ, but not necessarily was limited to the LLQ. Each bowel movement was recorded, and urgency, straining, feeling of incomplete evacuation, and Bristol stool form scale rating was recorded with each bowel movement. Normal stools were classified as a Bristol stool form scale rating of 3 to 5, hard stools as 1–2, and loose stools as 6–7 [14]. If the pain started the same hour that the individual passed stools it was recorded as pain associated with bowel movement. Similarly, if the last hour of the pain was the same hour that the participant passed stools it was registered as pain relieved by a bowel movement. The time of day when the subject first experienced pain was grouped into three categories: Morning from 4 to 12 am, afternoon from 12 noon to 8 pm, and night from 8 pm to 4 am.

### 2.4 Irritable Bowel Syndrome

IBS status was determined by the gastroenterologist during the consultation according to the Rome criteria used at that time (the Rome II criteria).

### 2.5 Statistical Analysis

Study group characteristics for the diary cohort in total and stratified by individuals with and without diverticulosis are presented in Table 1. Differences between individuals with and without diverticulosis with respect to dichotomous variables (sex, IBS status) was evaluated via the Pearson Chi‐Square test and differences in continuous variables (age) were evaluated via the Mann–Whitney test.

**TABLE 1:** Study group demographics in the total study population and separated according to the presence or absence of diverticulosis.

	Total	Non‐diverticulosis	Diverticulosis	*p*
Individuals, *n*	258	208	50	
Age, median (IQR)	56 (47–63)	53 (44–61)	62 (55–66)	< 0.001
Women, *n* (%)	166 (64)	136 (65)	30 (60)	0.733
IBS status by gastroenterologist, *n* (%)	48 (19)	40 (19)	8 (16)	0.936

Abbreviation: IBS, irritable bowel syndrome.

Diary symptom data for individuals with and without diverticulosis are presented in Table 2. Single observation variables included age, sex, IBS status, the occurrence of abdominal pain, the occurrence of LLQ pain, total number of abdominal pain episodes, and total number of pain episodes with pain located in the LLQ. Variables with more than one observation per person (Table 2) were pain severity, pain duration, the time of day of each abdominal pain episode (morning, afternoon, night), and Bristol stool scale, urgency, straining, and incompleteness ratings of each bowel movement. For variable with only a single observation per person, differences in dichotomous variables between individuals with and without diverticulosis were tested using logistic regression and were adjusted for age and sex. Differences in continuous variables between individuals with and without diverticulosis were tested using linear regression adjusted for age and sex. Analyses on variables with more than one observation per individual utilized mixed effect regression models with participant identity as a random intercept. Due to non‐normality in some variables, formal statistical inference was made via the nonparametric bootstrap with 2000 bootstrap repetitions for the linear regression models.

**TABLE 2:** Pain and stool characteristics stratified by individuals with and without diverticulosis.

	Total	Non‐diverticulosis	Diverticulosis	*p* ^b^
Individuals, *n*	258	208	50	
Pain at least once, *n* (%)	80 (31)	65 (31)	15 (30)	0.551
Average pains per person, *n*	1.05 (272/258)	1.07 (223/208)	0.98 (49/50)	0.490
LLQ pain at least once, *n* (%)	55 (21)	43 (21)	12 (24)	0.244
Average LLQ pain per person, *n*	0,62 (159/258)	0,59 (122/208)	0,74 (37/50)	0.491
Pain starts with BM, *n* (%)	41 (15)	29 (13)	12 (24)	0.068
Pain ends with BM, *n* (%)	13 (5)	7 (3)	6 (12)	0.137
Pain severity 1–3, mean (SD)	1.4 (0.6)	1.4 (0.5)	1.6 (0.7)	0.384
Pain duration (h), mean (SD)	4.3 (4.4)	4.5 (4.7)	3.3 (2.5)	0.139
Morning 04–12, *n* (%)	107 (39)	88 (39)	19 (39)	0.483
Afternoon 12–20, *n* (%)	101 (37)	85 (38)	16 (33)	0.779
Night 20–04, *n* (%)	64 (24)	50 (22)	14 (29)	0.362
Bowel movement, *n*	2372	1842	530	
BM per day & per person	1.31	1.27	1.51	0.098
Bristol stool scale, mean (SD)	4.1 (1.4)	4.1 (1.4)	4.1 (1.4)	0.350
Normal stools *n* (%)^a^	1669 (70)	1304 (71)	365 (69)	0.413
Hard stools *n* (%)^a^	319 (13)	245 (13)	74 (14)	0.393
Loose stools *n* (%)^a^	345 (17)	265 (16)	80 (17)	0.430
Urgency, *n* (%)	482 (24)	356 (22)	126 (28)	0.886
Straining, *n* (%)	584 (28)	456 (28)	128 (29)	0.404
Incompleteness, *n* (%)	984 (41)	777 (42)	207 (39)	0.716

Abbreviations: BM, bowel movement; LLQ, left lower quadrant.

^a^ On the Bristol stool scale: Normal stools 3–5, Hard 1–2, Loose 6–7. Only 233 individuals had information on the Bristol stool scale.

^b^ Differences in dichotomous variables between individuals with and without diverticulosis were tested using mixed effects logistic regression adjusted for age and sex. Differences in continuous variables between individuals with and without diverticulosis were tested using mixed effected linear regression adjusted for age and sex. Due to non‐normality in some variables, formal statistical inference was made via the nonparametric bootstrap with 2000 bootstrap repetitions.

A two‐tailed *p*‐value < 0.05 was considered statistically significant. No allowance for multiple statistical inference was made in this interpretation of statistical hypothesis tests. The sample size available yielded statistical power 0.8 at the 0.05 level of statistical significance (two‐tailed) for an odds ratio of 2.06 or greater which is commonly used as the minimum clinically important effect size. All analyses were performed using Stata 17 (StataCorp, College Station, TX).

## 3 Results

Descriptive information on the study population divided on diverticulosis status is presented in Table 1. Individuals with diverticulosis were on average older than those without diverticulosis but similar in terms of sex distribution and IBS status.

### 3.1 Abdominal Pain

A total of 272 pain episodes were registered in the diaries from 258 individuals. Those with diverticulosis were not more likely to experience abdominal pain than those without diverticulosis (OR 1.24, CI 0.61–2.55, *p* = 0.55). Start of pain with bowel movement, end of pain with bowel movement, and pain severity were not associated with diverticulosis (Table 2).

### 3.2 Left Lower Quadrant (LLQ) Abdominal Pain

Individuals with diverticulosis did not experience LLQ abdominal pain more often than those without diverticulosis (OR 1.59, CI 0.73–3.49, *p* = 0.24). However, when those with diverticulosis had pain, it was more often located in the LLQ (OR 2.45, CI 1.02–5.86, *p* = 0.045).

### 3.3 Bowel Movement

Diverticulosis was not associated with more frequent bowel movements, urgency, straining, feeling of incomplete evacuation, or a different Bristol Stool score (Table 2).

### 3.4 Time of Day

Abdominal pain did not start at different times during the day for those with diverticulosis compared with those without diverticulosis (Table 2).

## 4 Discussion

In this unique population‐based study, diverticulosis was not associated with more frequent abdominal pain or more LLQ abdominal pain overall in a community sample. However, when individuals with diverticulosis had abdominal pain, it was more frequently located in the LLQ compared with those without diverticulosis. Further, the duration and severity of abdominal pain and the stool form, were similar between those with and without diverticulosis.

One entity, termed symptomatic uncomplicated diverticular disease (SUDD) has been described and is defined as diverticulosis and chronic abdominal pain without colitis or diverticulitis [15]. There are reports in the literature that SUDD is clinically different from IBS by being characterized by more frequent and severe pain, as for example reported by Cuomo et al. [6] in a case control study. Age of onset may also separate “diverticulosis pain” and IBS as the onset of IBS is often earlier in adult life while diverticulosis usually develops at an older age [7]. Simpson et al. [8] observed in a non‐population‐based cohort study that individuals with diverticulosis showed two types of pain, 36% reporter shorter, recurrent pain, while 20% described longer pain often lasting over 24 h. Tursi et al. proposed that a longer duration of pain may be used to separate SUDD from IBS. They showed in a group of patients referred for colonoscopy that individuals with diverticulosis that had higher fecal calprotectin also had longer and more severe abdominal pain [3]. Another possible difference is that the pain in IBS is diffuse and poorly localized while pain in SUDD is suggested to be more localized in the LLQ [9]. However, none of these studies are population‐based and our results do not align with the hypothesis of SUDD.

Perry et al. [4] had similar results to ours when they compared 182 individuals with diverticulosis versus 128 without diverticulosis and found no association between diverticulosis and abdominal pain lasting over 24 h [4]. Larger population‐based studies are needed to shed more light on this subject, but such studies are challenging, as the diagnosis of diverticulosis is dependent on endoscopy which is why the current study is unique and important.

The prevalence of diverticulosis and IBS are both high, it is thus likely that these conditions coexist as suggested in a population‐based study by Jung et al. [5] There are some similarities between the two diagnoses, and they may share common pathophysiologic mechanisms, such as visceral hypersensitivity [15]. Our data shows a similar prevalence of IBS and similar odds of abdominal pain among individuals with and without diverticulosis, supporting the theory that pain in uncomplicated diverticulosis may indeed represent IBS. Although IBS status was determined by the gastroenterologist based on the current criteria at that time, there is little reason to think that the revised criteria would change the distribution of IBS between groups, as there was no difference in frequency of pain or stool habits in the diaries. Further, studies comparing Rome criteria (e.g., Rome II vs. Rome III) suggest only small differences in diagnostic accuracy [16].

Recall bias is a major limitation of retrospective questionnaire collected data [12]. The novelty and strength in this study is the prospectively collected diary data with detailed location of the abdominal pain. This methodology avoids recall bias associated with registration of symptoms retrospectively. We have previously shown that the pattern of symptom reports in diaries show a high reliability over time suggesting 7‐day diaries are sufficient to capture the overall symptom pattern in an individual [17]. It should be noted that the study design does not allow for any conclusions about causality in the association between presence of diverticulosis and pain in the LLQ in participants with pain. Even though it seems unlikely that the pain causes diverticula, the association may be explained by a third factor that is associated with both diverticular disease and LLQ pain in participants reporting pain.

The strength of the study is its population‐based prospective design, and that each individual underwent colonoscopy as well as registering prospectively abdominal symptoms for a whole week, which has not been done elsewhere. In addition, our data collection included registration of the location of the abdominal pain, is lacking in most previous studies [18]. Lastly the present study was population‐based thus minimizing selection bias.

The limitations of the current study include its modest sample size, which may have led to type II error in identifying cases of abdominal pain as well as missing data as not all those that did colonoscopy filled out the abdominal symptom diaries. The findings from the present study should be replicated in other geographical areas and cultures to extend the generalizability. Future studies could use imaging to improve the feasibility of collecting data on a larger population sample.

## 5 Conclusion

To conclude, in this study, diverticulosis was not associated with more frequent abdominal pain or LLQ abdominal pain when compared with those without diverticulosis. Bowel habit was not abnormal in diverticulosis. Our results do not support that uncomplicated diverticulosis causes symptoms in individuals without a history of acute diverticulitis.

## Author Contributions

**Bjarki T. Alexandersson:** data acquisition, data analysis, writing – original draft (lead), writing – review and editing (equal). **Michael P. Jones:** methodology, data curation, formal analysis, writing – review and editing (equal). **Anna Forsberg:** supervision, writing – review and editing (equal). **Charlotte R. H. Hedin:** supervision, writing – review and editing (equal). **Ellionore Järbrink‐Sehgal:** data curation, formal analysis, writing – review and editing (equal). **Susanna Walter:** methodology, investigation, writing – review and editing (equal). **Nicholas J. Talley:** conceptualization, methodology, investigation, writing – review and editing (equal). **Lars Agreus:** conceptualization, methodology, investigation, project administration, writing – review and editing (equal). **Anna Andreasson:** conceptualization, methodology, data curation, formal analysis, project administration, supervision, writing – review and editing (equal). **Peter T. Schmidt:** conceptualization, funding acquisition, methodology, supervision, writing – review and editing (lead).

## Conflicts of Interest

Nicholas J. Talley Norgine (2021) (IBS interest group), personal fees from Allakos (gastroduodenal eosinophilic disease) (2021), twoXAR Viscera Labs, (USA 2021) (IBS‐ diarrhoea), IsoThrive (2021) (esophageal microbiome), BluMaiden (microbiome advisory board) (2021), Rose Pharma (IBS) (2021), Intrinsic Medicine (2022) (human milk oligosaccharide), Comvita Mānuka Honey (2021) (digestive health), Astra Zeneca (2022), Biocodex (FD) (2024) outside the submitted work. In addition, Dr. Talley has a patent Nepean Dyspepsia Index (NDI) 1998, “Diagnostic marker for functional gastrointestinal disorders” Australian Provisional Patent Application 2021901692. “Methods and compositions for treating age‐related neurodegenerative disease associated with dysbiosis” US Application No. 63/537,725. Committees: NHMRC Principal Committee (Research Committee) Asia Pacific Association of Medical Journal Editors, Rome V Working Team Member (Gastroduodenal Committee), International Plausibility Project Co‐Chair (Rome Foundation funded). Community group: Advisory Board, IFFGD (International Foundation for Functional GI Disorders), AusEE. Editorial: Mayo Clinic Proceedings (Assoc Ed), Up to Date (Section Editor), Precision and Future Medicine, Sungkyunkwan University School of Medicine, South Korea, Med (Journal of Cell Press). Dr. Talley is supported by funding from the National Health and Medical Research Council (NHMRC) to the Centre for Research Excellence in Digestive Health and he holds an NHMRC Investigator grant. C. R. H. Hedin served as a speaker and/or advisory board member for AstraZeneca, Abbvie, Dr. Falk Pharma and the Falk Foundation, Galapagos, Janssen, Pfizer, Ferring, Takeda, Tillotts Pharma, and received grant support from Tillotts and Takeda. Peter T Schmidt has served as Advisory Board member for Gilead Nordic, Janssen Cilag, and Norgine.

## Data Availability

The data that support the findings of this study are available on request from the corresponding author. The data are not publicly available due to privacy or ethical restrictions.
